# Plant-mediated links between detritivores and aboveground herbivores

**DOI:** 10.3389/fpls.2013.00380

**Published:** 2013-09-24

**Authors:** Susanne Wurst

**Affiliations:** Dahlem Centre of Plant Sciences, Functional Biodiversity, Freie Universität BerlinBerlin, Germany

**Keywords:** above–belowground interactions, earthworms, Collembola, microorganisms, herbivores, defense, priming

## Abstract

Most studies on plant-mediated above–belowground interactions focus on soil biota with direct trophic links to plant roots such as root herbivores, pathogens, and symbionts. Detritivorous soil fauna, though ubiquitous and present in high abundances and biomasses in soil, are under-represented in those studies. Understanding of their impact on plants is mainly restricted to growth and nutrient uptake parameters. Detritivores have been shown to affect secondary metabolites and defense gene expression in aboveground parts of plants, with potential impacts on aboveground plant–herbivore interactions. The proposed mechanisms range from nutrient mobilization effects and impacts on soil microorganisms to defense induction by passive or active ingestion of roots. Since their negative effects (disruption or direct feeding of roots) may be counterbalanced by their overall beneficial effects (nutrient mobilization), detritivores may not harm, but rather enable plants to respond to aboveground herbivore attacks in a more efficient way. Both more mechanistic and holistic approaches are needed to better understand the involvement of detritivores in plant-mediated above–belowground interactions and their potential for sustainable agriculture.

## INTRODUCTION

Links between the below- and the aboveground compartments of terrestrial ecosystem have been the focus of a growing number of studies in the last two decades. Integrating the two compartments is crucial for a better understanding of the ecology, function, and evolution of terrestrial ecosystems. In the first review articles on plant-mediated effects of soil biota on aboveground herbivores (Scheu and Setälä, 2001; Scheu and Setälä, 2001; Wardle et al., 2004) soil organisms were divided in two groups, either with or without direct trophic links to the plant. The majority of studies and reviews that have followed focused on soil biota with direct trophic interaction to plant roots such as root herbivores, pathogens, and arbuscular mycorrhizal fungi (reviewed by, e.g., Bezemer and van Dam, 2005; Koricheva et al., 2009; van Dam and Heil, 2011; Johnson et al., 2012). Soil organisms in indirect interaction with roots, such as members of the decomposer subsystem, have received far less attention (but see Wurst, 2010). This is surprising, since detritivores are ubiquitous and very abundant in soil, play a crucial role in nutrient turnover and decomposition processes, and can have profound effects on plant and herbivore performance mediated by different mechanisms.

In this review, I focus on plant-mediated links between detritivorous soil fauna and aboveground herbivores. With earlier reviews having focused mainly on soil biota with direct trophic links to plant roots, this review specifically deals with soil biota in indirect interaction with roots. First, I give a short summary of the reported impacts of detritivores on aboveground herbivores (see Plant-Mediated Effects of Detritivores on Aboveground Herbivores). Then I present investigated and proposed mechanisms behind the observed effects (see Mechanisms of How Detritivores Affect Plant and Aboveground Herbivore Performance), before comparing the mechanisms involved in the effects of soil biota in indirect interaction vs. direct interaction with roots (see Mechanisms Involved in the Effects of Soil Biota in Direct and Indirect Interaction with Roots). Finally, I highlight promising avenues for future research (see Research Perspectives). With this review, I hope to place detritivores “in the spot light,” because their rather neglected role in studies on plant-mediated interactions between soil biota and aboveground herbivores and their antagonists does not reflect their importance in below–aboveground links of terrestrial ecosystems.

## PLANT-MEDIATED EFFECTS OF DETRITIVORES ON ABOVEGROUND HERBIVORES

One of the first studies of plant-mediated effects of detritivores on aboveground herbivores was carried out on collembolans and earthworms (Scheu et al., 1999). The effects of Collembola (*Heteromurus nitidus* and *Onychiurus scotarius*) and endogeic earthworms (*Aporrectodea caliginosa* and *Octolasion tyrtaeum*) on growth of a grass (*Poa annua*) and a legume (*Trifolium repens*), and on aphid (*Myzus persicae*) reproduction on their leaves were investigated. Besides effects on plant growth, Collembola had strong effects on aphid reproduction that differed in direction and magnitude between the different plant species. On average, aphid reproduction was decreased by 45% on *T. repens*, but transiently increased ca. threefold on *Poa annua* in presence of Collembola. Earthworms had a strong positive, but also transient effect on aphid reproduction on both plant species. Subsequent studies investigated the impacts of earthworms (Bonkowski et al., 2001; Wurst and Jones, 2003; Wurst et al., 2003, 2004a, b; Newington et al., 2004; Poveda et al., 2005; Eisenhauer et al., 2010; Wurst and Forstreuter, 2010; Johnson et al., 2011) and Collembola (Haase et al., 2008; Ke and Scheu, 2008; Schütz et al., 2008) on aboveground herbivores. In the majority of studies, the impact of earthworms and Collembola was investigated on aboveground phloem-feeding aphids. As far as we are aware, only one study (Newington et al., 2004) investigated the plant-mediated effects of earthworms on a leaf-chewing caterpillar (*Mamestra brassicae*). When considering also studies on the impact of detritivores on plant communities, two studies, as far as we are aware, investigated the effects of earthworms on herbivorous snails (Thompson et al., 1993; Wurst and Rillig, 2011). Thus, the majority of studies focused on one feeding type of aboveground herbivores (phloem-feeders), and earthworms and Collembola as detritivores.

The plant-mediated effects of detritivores on aboveground herbivores vary in strength and direction. The impacts of earthworms on above- and belowground herbivores have been reviewed before (Wurst, 2010). In short, their plant-mediated effects on aboveground herbivores range from negative, neutral to positive, and may depend on soil characteristics such as soil nutrient content, distribution of soil organic matter, and the soil microbial community. The effects of Collembola on aphids also range from negative through neutral to positive and were suggested to differ in direction between more and less palatable plant species (Scheu et al., 1999) and to be affected by fertilizer addition (Schütz et al., 2008). 

Thus the effects of detritivores on plant–herbivore interactions are highly context dependent and are influenced by soil and plant characteristics. Overall, it is important to note that the effects of the detritivores on aboveground herbivores are not always positive and related to an enhanced nutrient availability for the plants, but can also be negative suggesting other mechanisms besides nutrient mobilization. Mechanisms by which detritivores may affect plant and herbivore performance are discussed in the next section.

## MECHANISMS OF HOW DETRITIVORES AFFECT PLANT AND ABOVEGROUND HERBIVORE PERFORMANCE

Soil organisms of the decomposer subsystem (Wardle et al., 2004) are responsible for important ecological processes such as litter incorporation, litter fragmentation, nutrient mineralization and immobilization which affect the nutrient availability for plants with consequences for herbivore performance. These processes are interactively performed by functionally different soil organisms belonging to a variety of size classes, ranging from microbes to macrofauna (Bardgett, 2005; Wurst et al., 2012). Thus, effects of detritivorous soil organisms on plant and aboveground herbivore performance are often mediated by their impacts on nutrient turnover and decomposition processes. Other proposed mechanisms for their effects involve grazing on soil biota in direct interaction with plants (e.g., mycorrhizal fungi and root pathogens), dispersal of microorganisms, changes of soil structure and hormone-like effects (reviewed by Scheu and Setälä, 2001; Scheu and Setälä, 2001). All these processes can affect nutrient availability and growth of plants with consequences on aboveground herbivores.

It has, however, also been shown that detritivorous soil organisms can affect defense compounds in plants and the expression of defense-related genes. It has been documented that earthworms can reduce aphid reproduction (Wurst et al., 2003) and that this may be related to changes in plant defense compounds induced by earthworms (Wurst et al., 2004a, b). Earthworms enhanced the concentration of phytosterols, but this effect was influenced by the litter distribution in soil (Wurst et al., 2004a), and decreased the concentration of the iridoid glycoside catapol in leaves of *Plantago lanceolata* (Wurst et al., 2004b). Both compounds play an important role for herbivore performance; phytosterols are precursors of molting hormones, while iridoid glycosides are secondary metabolites known to deter generalist insect herbivores and pathogens. Additionally, effects of earthworms on the N-based secondary metabolites glucosinolates in Brassicaceae were documented; however, the effects varied between different groups of glucosinolates. While sulfur-containing glucosinolates were reduced by earthworms in *Brassica oleracea* leaves (Wurst et al., 2006), aliphatic glucosinolates were reduced and aromatic glucosinolates were enhanced in *Sinapis alba* (Lohmann et al., 2009). Blouin et al. (2005) showed that earthworms can change stress-responsive gene expression (such as genes coding for lipoxygenase and cysteine protease) and make rice (*Oryza sativa*) plants more resistant to root-feeding nematodes.

Recently, it has been documented that earthworms influence the expression of genes involved in cell proliferation and stress response in the model plant *Arabidopsis thaliana* (Jana et al., 2010). Puga-Freitas et al. (2012) proposed that signal molecules (such as indole acetic acid, IAA) may mediate the earthworm effects on plant growth. They also showed that earthworms change the expression of genes responsive to abiotic and biotic stress, and to the application of exogenous hormones. Since the plant responses to earthworms resembled responses known to occur in the systemic resistance induced by plant growth promoting rhizobacteria (PGPR; induced systemic resistance, ISR) and/or pathogens (systemic acquired resistance, SAR), the latter authors and Wurst (2010) proposed that earthworm effects on plant resistance may be mediated by changes in abundance or activity of rhizosphere microorganisms such as PGPR. In summary, experimental studies document that earthworms systemically affect secondary metabolites and the expression of stress-related genes in different plant species which may mediate their effect on aboveground herbivore performance.

For Collembola, it has been recently reported that they also have the ability to affect the expression of defense-related and auxin-responsive genes (Endlweber et al., 2011). The latter authors therefore suggested that Collembola improve plant resistance against herbivores by enhancing the production of secondary compounds while concomitantly compensating the production costs by fostering root growth and nutrient exploitation.

**FIGURE 1:**
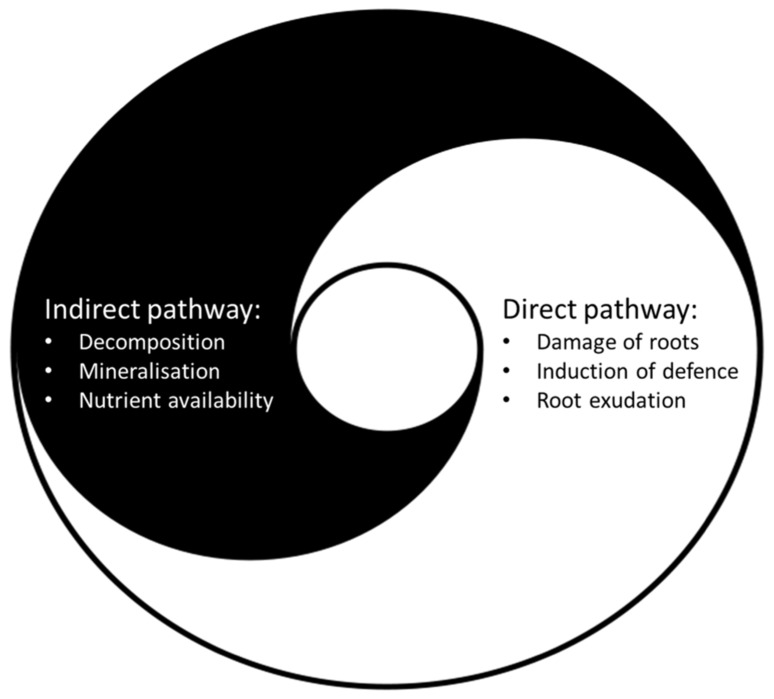
**The indirect and the direct pathway to plants are intimately linked and many soil biota affect both pathways.** Soil biota in indirect interaction with roots (e.g., detritivores) enhance nutrient availability by promoting decomposition and mineralization processes (indirect pathway), but also damage roots which may lead to induction of defense and root exudation (direct pathway). Soil biota in direct trophic interaction with roots (e.g., insect root herbivores) feed and damage roots, induce defense, and change root exudation (direct pathway) which may lead to enhanced microbial activity, mineralization, and nutrient availability (indirect pathway) [image: modified from Christian Hummert (Ixitixel)].

This duality of impact on plant growth and defense may be a characteristic feature of the impact of detritivores on plants (**Figure 1**). As well as enhancing nutrient availability for the plants, they also induce stress-responsive genes and systemically change the production of secondary metabolites in the plants. Since their beneficial effects (nutrient mobilization) may counterbalance their negative effects (disruption or direct feeding of roots), detritivores may not harm, but rather prepare plants to respond to aboveground herbivore attacks in a more efficient way. This issue is further developed in Section “Research Perspectives” regarding future research perspectives.

## MECHANISMS INVOLVED IN THE EFFECTS OF SOIL BIOTA IN DIRECT AND INDIRECT INTERACTION WITH ROOTS

The impact on shoot herbivores by soil biota with a direct trophic link to the plant can be mediated by changes in water and nutrient uptake and/or an induction of plant defense that affects the whole plant systemically (reviewed by van Dam et al., 2003; Bezemer and van Dam, 2005; Koricheva et al., 2009; van Dam and Heil, 2011; Johnson et al., 2012). The same seems to be true for soil biota in indirect interaction to plants as summarized above for detritivores such as earthworms and Collembola. Interestingly, the Collembola *Protaphorura* *fimata* has been reported to act as a root herbivore and may predominately feed on roots (Endlweber et al., 2009). Other well-known detritivores, the crustacean isopods *Porcellio* *scaber* and *Armadillidium vulgare*, can become herbivores on jasmonate-deficient plants (Farmer and Dubugnon, 2009). A recent study reported that the anecic earthworm *Lumbricus* *terrestris* can act as a shoot herbivore (Griffith et al., 2013). These studies demonstrate that detritivorous soil biota may be more omnivorous than previously thought and that living plant material might be a substantial part of their diet. Besides the challenge to classify soil biota into distinct feeding guilds, the interaction of soil biota with plants generally has both a direct and indirect component. For example, feeding by root herbivores may induce plant defense and root exudation (direct pathway) leading to a stimulation of the microbial activity and mineralization processes (indirect pathway) (Bardgett et al., 1999; Dawson et al., 2004). Thus soil biota in direct trophic interaction also change indirect pathways to plants. On the other hand, soil biota with mainly indirect non-trophic interaction to plants may affect soil biota in direct interaction with roots, disturb the root system and thus also affect the direct pathway to plants (**Figure 1**). The relative contribution of soil biota to the indirect and the direct pathway may also change with conditions, such as the abundance and quality of roots, the microbial community and the nutrient availability in soil.

## RESEARCH PERSPECTIVES

Our knowledge on effects of detritivores on plant–herbivore interactions above the ground are almost exclusively based on earthworms and collembolan studies. Other detritivorous soil fauna belonging to taxonomic groups such as isopoda, diplopoda, or insects have been not or very rarely considered. González-Megías and Müller (2010) investigated the effects of a detritivorous beetle larvae (*Morica hybrida*, Tenebrionidae) in interaction with a belowground herbivorous beetle larvae (*Cebrio gypsicola*, Cebrionidae) and aboveground herbivores on *Moricandia moricandioides* (Brassicaceae) and aboveground higher trophic levels in the field. They reported effects of detritivores up to the third trophic level above the ground: parasitoid attack rate and abundance were positively affected by the presence of detritivores. This is one of the few studies (Wurst and Jones, 2003; Poveda et al., 2005; González-Megías and Müller, 2010; Johnson et al., 2011) that followed the effects of detritivores up to the third level, i.e., the antagonists of herbivores above the ground. The results show that detritivores cannot only change direct defense of plants against herbivores, but may influence indirect defense mechanisms such as the recruitment of herbivore antagonists. As far as I am aware, there are no studies on the impacts of isopoda and diplopoda on plant–herbivore interactions and direct or indirect plant defense above the ground. In general, studies with different detritivore taxa and more field studies are needed to better judge the plant-mediated impact of detritivores on higher trophic levels and indirect plant defense mechanisms.

Besides the claim for a more holistic approach that involves also studying higher trophic levels under natural conditions, it is important to further elucidate the underlying mechanisms of plant-mediated interactions between detritivores, aboveground herbivores, and their antagonists. Here, it is promising to ask whether detritivores can prime plants to better cope with stresses. Priming of plants for a more efficient activation of defense responses has been documented for PGPR (Pieterse et al., 2003; Conrath et al., 2006; van Loon, 2007). By inducing stress-responsive genes and counterbalancing the costs through an enhanced nutrient mobilization and availability for the plants, detritivores may also prime plants to be better prepared for abiotic and biotic stresses. They may do this either directly or mediated by changes in the soil microbial community, e.g., the abundance of PGPR (Wurst, 2010). All this is so far unknown, but waits to be explored in future research that may also contribute to the development of more sustainable plant protection strategies.

In summary, I propose two promising approaches for future research based on the described knowledge gaps and the potential importance of detritivores for plant-mediated above–belowground interactions: on the one hand, more holistic approaches including different detritivorous taxa studied under (semi-) natural conditions to determine their plant-mediated impacts on herbivores and their antagonists in natural and agricultural systems; on the other hand, more mechanistic studies addressing the underlying mechanisms with special emphasis on effects of detritivores on plant physiology and defense pathways. Both approaches combined will help to better understand the impact of detritivores on aboveground plant herbivore interactions and to evaluate their potential for improving crop yield and herbivore resistance in sustainable agriculture.

## Conflict of Interest Statement

The author declares that the research was conducted in the absence of any commercial or financial relationships that could be construed as a potential conflict of interest.

## References

[B1] Bardgett R. D. (2005). *The Biology of Soil. A Community and Ecosystem Approach.* Oxford: Oxford University Press. 10.1093/acprof:oso/9780198525035.001.0001

[B2] Bardgett R. D. Denton C. S. Cook R. (1999). Below-ground herbivory promotes soil nutrient transfer and root growth in grassland. *Ecol. Lett.* 2 357–360 10.1046/j.1461-0248.1999.00001.x

[B3] Bezemer T. M van Dam N. M. (2005). Linking aboveground and belowground interactions via induced plant defenses. *Trends Ecol. Evol.* 20 617–624 10.1016/j.tree.2005.08.006 16701445

[B4] Blouin M. Zuily-Fodil Y. Pham-Thi A. T. Laffray D. Reversat G. Pando A. (2005). Belowground organism activities affect plant aboveground phenotype, inducing plant tolerance to parasites. *Ecol. Lett.* 8 202–208 10.1111/j.1461-0248.2004.00711.x

[B5] Bonkowski M. Geoghegan I. E. Birch A. N. E. Griffiths B. S. (2001). Effects of soil decomposer invertebrates (protozoa and earthworms) on an above-ground phytophagous insect (cereal aphid) mediated through changes in the host plant. *Oikos* 95 441–450 10.1034/j.1600-0706.2001.950309.x

[B6] Conrath U. Beckers G. J. M. Flors V. Garcia-Agustin P. Jakab G. Mauch F. (2006). Priming: getting ready for battle. *Mol. Plant Microbe Interact.* 19 1062–1071 10.1094/MPMI-19-1062 17022170

[B7] Dawson L. A. Grayston S. J. Murray P. J. Ross J. M. Reid E. J. Treonis A. M. (2004). Impact of *Tipula paludosa* larvae on plant growth and the soil microbial community. *Appl. Soil Ecol.* 25 51–61 10.1016/S0929-1393(03)00099-4

[B8] Eisenhauer N. Hoersch V. Moeser J. Scheu S. (2010). Synergistic effects of microbial and animal decomposers on plant and herbivore performance. *Basic Appl. Ecol.* 11 23–34 10.1016/j.baae.2009.11.001

[B9] Endlweber K. Krome K. Welzl G. Schaeffner A. R. Scheu S. (2011). Decomposer animals induce differential expression of defence and auxin-responsive genes in plants. *Soil Biol. Biochem.* 43 1130–1138 10.1016/j.soilbio.2010.11.013

[B10] Endlweber K. Ruess L. Scheu S. (2009). Collembola switch diet in presence of plant roots thereby functioning as herbivores. *Soil Biol. Biochem.* 41 1151–1154 10.1016/j.soilbio.2009.02.022

[B11] Farmer E. E. Dubugnon L. (2009). Detritivorous crustaceans become herbivores on jasmonate-deficient plants. *Proc. Natl. Acad. Sci. U.S.A.* 106 935–940 10.1073/pnas.0812182106 19139394 PMC2630106

[B12] González-Megías A. Müller C. (2010). Root herbivores and detritivores shape aboveground multitrophic assemblage through plant-mediated effects. *J. Anim. Ecol.* 79 923–931 10.1111/j.1365-2656.2010.01681.x 20302605

[B13] Griffith B. Tuerke M. Weisser W. W. Eisenhauer N. (2013). Herbivore behavior in the anecic earthworm species *Lumbricus terrestris* L.? *Eur. J. Soil Biol.* 55 62–65 10.1016/j.ejsobi.2012.12.002

[B14] Haase J. Brandl R. Scheu S. Schaedler M. (2008). Above- and belowground interactions are mediated by nutrient availability. *Ecology* 89 3072–3081 10.1890/07-1983.1 31766818

[B15] Jana U. Barot S. Blouin M. Lavelle P. Laffray D. Repellin A. (2010). Earthworms influence the production of above- and belowground biomass and the expression of genes involved in cell proliferation and stress responses in *Arabidopsis thaliana*. *Soil Biol. Biochem.* 42 244–252 10.1016/j.soilbio.2009.10.022

[B16] Johnson S. N. Clark K. E. Hartley S. E. Jones T. H. McKenzie S. W. Koricheva J. (2012). Aboveground-belowground herbivore interactions: a meta-analysis. *Ecology* 93 2208–2215 10.1890/11-2272.1 23185882

[B17] Johnson S. N. Staley J. T. McLeod F. A. L. Hartley S. E. (2011). Plant-mediated effects of soil invertebrates and summer drought on above-ground multitrophic interactions. *J. Ecol.* 99 57–65 10.1111/j.1365-2745.2010.01748.x

[B18] Ke X. Scheu S. (2008). Earthworms, Collembola and residue management change wheat (*Triticum aestivum*) and herbivore pest performance (Aphidina: *Rhophalosiphum padi*). *Oecologia* 157 603–617 10.1007/s00442-008-1106-y 18654802

[B19] Koricheva J. Gange A. C. Jones T. (2009). Effects of mycorrhizal fungi on insect herbivores: a meta-analysis. *Ecology* 90 2088–2097 10.1890/08-1555.1 19739371

[B20] Lohmann M. Scheu S. Mueller C. (2009). Decomposers and root feeders interactively affect plant defence in *Sinapis alba*. *Oecologia* 160 289–298 10.1007/s00442-009-1306-0 19252930 PMC3085730

[B21] Newington J. E. Setala H. Bezemer T. M. Jones T. H. (2004). Potential effects of earthworms on leaf-chewer performance. *Funct. Ecol.* 18 746–751 10.1111/j.0269-8463.2004.00888.x

[B22] Pieterse C. M. J. Van Pelt J. A. Verhagen B. W. M. Ton J. Van Wees S. C. M. Leon-Kloosterziel K. M. (2003). Induced systemic resistance by plant growth-promoting rhizobacteria. *Symbiosis* 35 39–54

[B23] Poveda K. Steffan-Dewenter I. Scheu S. Tscharntke T. (2005). Effects of decomposers and herbivores on plant performance and aboveground plant-insect interactions. *Oikos* 108 503–510 10.1111/j.0030-1299.2005.13664.x

[B24] Puga-Freitas R. Barot S. Taconnat L. Renou J.-P. Blouin M. (2012). Signal molecules mediate the impact of the earthworm *Aporrectodea caliginosa* on growth, development and defence of the plant *Arabidopsis thaliana*. *PLoS ONE* 7:e49504. 10.1371/journal.pone.0049504 PMC3513312 23226498

[B25] Scheu S. (2001). Plants and generalist predators as links between the below-ground and above-ground system. *Basic Appl. Ecol.* 2 3–13 10.1078/1439-1791-00031

[B26] Scheu S. Setälä H. (2001). “Multitrophic interactions in decomposer food-webs,” in *Multitrophic Level Interactions* eds Tscharntke T. Bradford A. H. (Cambridge: Cambridge University Press) 223–264

[B27] Scheu S. Theenhaus A. Jones T. H. (1999). Links between the detritivore and the herbivore system: effects of earthworms and Collembola on plant growth and aphid development. *Oecologia* 119 541–551 10.1007/s004420050817 28307712

[B28] Schütz K. Nagel P. Dill A. Scheu S. (2008). Structure and functioning of earthworm communities in woodland flooding systems used for drinking water production. *Appl. Soil Ecol.* 39 342–351 10.1016/j.apsoil.2008.02.002

[B29] Thompson L. Thomas C. D. Radley J. M. A. Williamson S. Lawton J. H. (1993). The effect of earthworms and snails in a simple plant community. *Oecologia* 95 171–178 10.1007/BF00323487 28312939

[B30] van Dam N. M. Harvey J. A. Wackers F. L. Bezemer T. M. van der Putten W. H Vet L. E. M. (2003). Interactions between aboveground and belowground induced responses against phytophages. *Basic Appl. Ecol.* 4 63–77 10.1078/1439-1791-00133

[B31] van Dam N. M. Heil M. (2011). Multitrophic interactions below and above ground: en route to the next level. *J. Ecol.* 99 77–88 10.1111/j.1365-2745.2010.01761.x

[B32] van Loon L. C. (2007). Plant responses to plant growth-promoting rhizobacteria. *Eur. J. Plant Pathol.* 119 243–254 10.1007/s10658-007-9165-1

[B33] Wardle D. A. Bardgett R. D. Klironomos J. N. Setala H. van der Putten W. H. Wall D. H. (2004). Ecological linkages between aboveground and belowground biota. *Science* 304 1629–1633 10.1126/science.1094875 15192218

[B34] Wurst S. (2010). Effects of earthworms on above- and belowground herbivores. *Appl. Soil Ecol.* 45 123–130 10.1016/j.apsoil.2010.04.005

[B35] Wurst S. De Deyn G. B. Orwin K. (2012). “Soil Biodiversity and Functions”, in *Soil Ecology and Ecosystem Services* ed. Wall D. H. (Oxford: Oxford University Press) 28–45

[B36] Wurst S. Dugassa-Gobena D. Scheu S. (2004a). Earthworms and litter distribution affect plant-defensive chemistry. *J. Chem. Ecol.* 30 691–701 10.1023/B:JOEC.0000028425.43869.b8 15260217

[B37] Wurst S. Dugassa-Gobena D. Langel R. Bonkowski M. Scheu S. (2004b). Combined effects of earthworms and vesicular–arbuscular mycorrhizas on plant and aphid performance. *New Phytol.* 163 169–176 10.1111/j.1469-8137.2004.01106.x 33873788

[B38] Wurst S. Forstreuter M. (2010). Colonization of *Tanacetum vulgare* by aphids is reduced by earthworms. *Entomol. Exp. Appl.* 137 86–92 10.1111/j.1570-7458.2010.01035.x

[B39] Wurst S. Jones T. H. (2003). Indirect effects of earthworms (*Aporrectodea caliginosa*) on an above-ground tritrophic interaction. *Pedobiologia* 47 91–97 10.1078/0031-4056-00173

[B40] Wurst S. Langel R. Reineking A. Bonkowski M. Scheu S. (2003). Effects of earthworms and organic litter distribution on plant performance and aphid reproduction. *Oecologia* 137 90–96 10.1007/s00442-003-1329-x 12844255

[B41] Wurst S. Langel R. Rodger S. Scheu S. (2006). Effects of belowground biota on primary and secondary metabolites in *Brassica oleracea*. *Chemoecology* 16 69–73 10.1007/s00049-005-0328-2

[B42] Wurst S. Rillig M. C. (2011). Additive effects of functionally dissimilar above- and belowground organisms on a grassland plant community. *J. Plant Ecol.* 4 221–227 10.1093/jpe/rtr012

